# Global Diversity Hotspots and Conservation Priorities for Sharks

**DOI:** 10.1371/journal.pone.0019356

**Published:** 2011-05-05

**Authors:** Luis O. Lucifora, Verónica B. García, Boris Worm

**Affiliations:** 1 Consejo Nacional de Investigaciones Científicas y Técnicas (CONICET), Centro de Investigaciones Ecológicas Subtropicales (CIES), Centro de Investigaciones del Bosque Atlántico (CeIBA), Puerto Iguazú, Misiones, Argentina; 2 Department of Biology, Dalhousie University, Halifax, Nova Scotia, Canada; University of Zurich, Switzerland

## Abstract

Sharks are one of the most threatened groups of marine animals, as high exploitation rates coupled with low resilience to fishing pressure have resulted in population declines worldwide. Designing conservation strategies for this group depends on basic knowledge of the geographic distribution and diversity of known species. So far, this information has been fragmented and incomplete. Here, we have synthesized the first global shark diversity pattern from a new database of published sources, including all 507 species described at present, and have identified hotspots of shark species richness, functional diversity and endemicity from these data. We have evaluated the congruence of these diversity measures and demonstrate their potential use in setting priority areas for shark conservation. Our results show that shark diversity across all species peaks on the continental shelves and at mid-latitudes (30–40 degrees N and S). Global hotspots of species richness, functional diversity and endemicity were found off Japan, Taiwan, the East and West coasts of Australia, Southeast Africa, Southeast Brazil and Southeast USA. Moreover, some areas with low to moderate species richness such as Southern Australia, Angola, North Chile and Western Continental Europe stood out as places of high functional diversity. Finally, species affected by shark finning showed different patterns of diversity, with peaks closer to the Equator and a more oceanic distribution overall. Our results show that the global pattern of shark diversity is uniquely different from land, and other well-studied marine taxa, and may provide guidance for spatial approaches to shark conservation. However, similar to terrestrial ecosystems, protected areas based on hotspots of diversity and endemism alone would provide insufficient means for safeguarding the diverse functional roles that sharks play in marine ecosystems.

## Introduction

To derive a global conservation strategy for higher taxa (i.e. above the species level) it is fundamental to know where different species occur and which geographic areas harbour high species richness, concentrations of endemic or threatened species, or unique communities of species [1]. Increasing evidence, both in marine and terrestrial environments, shows that so-called ‘hotspots’ of total species richness are not always concordant with hotspots of endemism or threat and that concentrations of threatened species or local endemics may also occur in areas of lower richness [2], [3]. Therefore, a conservation strategy cannot be based solely on the hotspot approach, but needs to consider other biogeographic units in order to protect the full range of biodiversity [4].

Sharks are one of the most threatened groups of marine animals worldwide. According to a recent global assessment, 830,000 tonnes of sharks and rays are reportedly landed each year and landings are increasing steadily at an approximate rate of 2% annually [5]. Earlier studies estimated that 60 million individuals [6] or 1.35 million tonnes [7] of sharks and rays are killed globally, either in target fisheries or as unintended bycatch. These numbers were based primarily on reported catches, and may still be underestimates considering that the burgeoning shark fin trade alone involved a minimum of 26 to 73 million sharks per year in the late 1990s [8], with a combined weight of 1.7 million tonnes per year (median estimate). This volume has recently been increasing at an annual rate of 5.4% [9]. High exploitation rates coupled with a slow life history and low resilience to fishing mortality have resulted in population declines, particularly of large sharks, worldwide [e.g. 10]–[17] and pose a serious threat for the survival of many species. Several management strategies, such as setting maximum allowable catches and size limits, or closing of particular areas, have been developed to conserve individual species of sharks, particularly highly commercial ones [5], [18]–[20]. Yet, as these measures are often restricted to particular species, they may not offer a general framework for conserving the full diversity of sharks. A geographic conservation approach, in contrast, may include a wide variety of species of different ecological characteristics in a given area. Such an approach would require knowledge of the geographic distribution of all species in order to determine the distribution of shark diversity and endemicity and the levels of congruence between these two measures of biodiversity. In addition, an important first step is to assess the potential usefulness of a geographic approach, since it might require large enforcement efforts depending on the extension of the areas to protect. This information is so far lacking for sharks.

Global patterns of marine diversity have been, so far, studied in groups with high habitat specialization, resulting in conflicting patterns. For example, corals and coral-reef fauna peak in the tropics [2], [21]–[23], while highly pelagic tunas and billfish [24]–[26] and planktonic foraminifera [27] tend to reach maximum diversity at mid-latitudes. Studies that include multiple taxa have been conducted on a more restricted geographic scale. In the Western Central Pacific, an equatorial center of diversity has been reported in the Philippines, although two secondary peaks of diversity off Taiwan and Eastern Australia, on the edges of the study area, were also detected [28], coinciding with the patterns found for tunas and billfishes [24]–[26]. Sharks offer a good model to elucidate patterns of marine diversity because they are widely distributed in the world's oceans, inhabit a variety of habitats and have a manageable number of described species (507), as compared with marine bony fishes (>15,000 spp.) or invertebrates (>100,000 spp.).

Here we (1) determine global richness and endemism hotspots for sharks and evaluate their usefulness for area prioritization for conservation; (2) compare global shark richness hotspots with hotspots for species threatened by the fin trade; (3) determine global hotspots for shark functional diversity – as measured by the richness of shark ecomorphotypes (see methods) – and (4) discuss conservation measures required to preserve sharks at the global scale.

## Results

**Figure 1:**
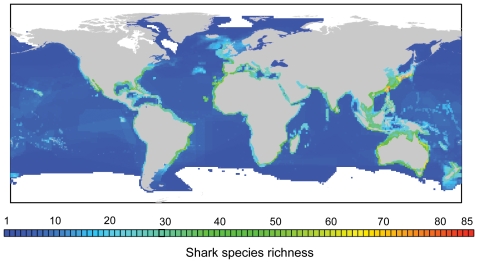
Global pattern of total shark species richness. The map indicates the number of shark species present in each cell of 1° longitude by 1° latitude. Richness hotspots of >50 shark species are coloured in bright green, yellow and red.

**Figure 2:**
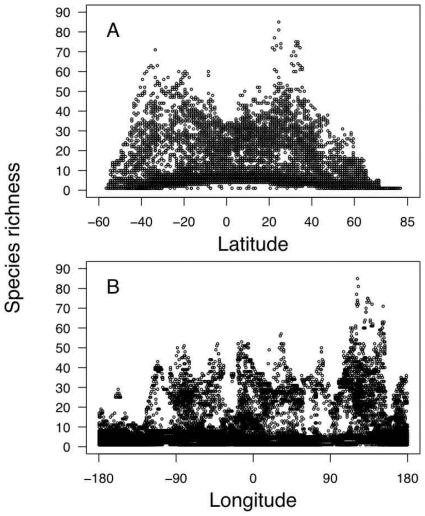
Relationship of shark species richness with (A) latitude and (B) longitude. Negative numbers indicate latitude south or longitude west.

Shark species richness was found to be highest on continental shelves and markedly lower in the open ocean (Fig. 1). Latitudinal patterns showed a bimodal distribution of species richness peaking between 30 and 40 degrees of latitude in both hemispheres (Fig. 2A). Also, for the same latitude, the Western Pacific Ocean harboured more species per cell than any other part of the world (Fig. 2B); in particular the region from Southern Japan to Southern Australia showed the highest richness of sharks worldwide (Fig. 1). Individual hotspots of species richness were located off Southern Japan and Taiwan (up to 85 species per 1°×1° cell) and off Northeast and Southeast Australia (up to 74 species per cell) (Fig. 1).

**Figure 3:**
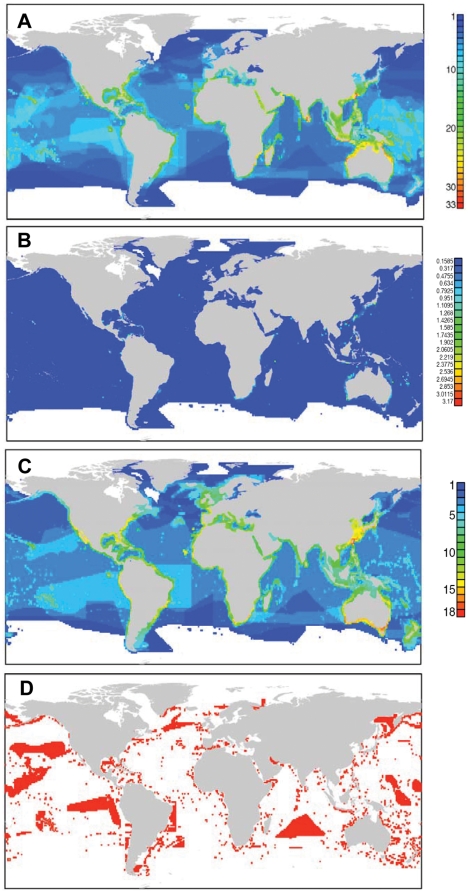
Shark conservation priorities. (A) Richness pattern of 52 shark species affected by the shark fin trade. (B) Pattern of shark endemism, quantified as the sum of the inverse of the geographic range size of all species present in the cell. (C) Pattern of shark functional richness, quantified as the number of shark ecomorphotypes (as defined by Compagno 1990) present in the cell. (D) Priority areas for shark conservation; each area was selected because it contains either the top 5% of species richness or endemism for each of 93 major shark biogeographic units (areas characterized by the presence of a unique set of species as identified by a cluster analysis).

Species included in the shark fin trade (n = 52) include large coastal carcharhinids such as Grey reef (*Carcharhinus amblyrhynchos*) and Bull shark, (*C. leucas*), coastal-pelagic species such as Great white (*Carcharodon carcharias*) and Hammerhead sharks (*Sphyrna* spp.), and oceanic species such as Oceanic whitetip (*C. longimanus*) and Thresher sharks (*Alopias* spp.), among others. Many of these species are recognized as globally threatened by the International Union for the Conservation of Nature (see www.iucnredlist.org), and represent an urgent conservation priority [8]–[9], [12]–[13], [19]–[20]. Species included in the shark fin trade, however, showed markedly different patterns of diversity when compared to all shark species. Areas of high richness of fin-trade species were broader in geographic extent, located closer to the Equator, and extended more towards open-ocean areas, reflecting a high proportion of pelagic species (Fig. 3A).

Centers of shark species endemism (Fig. 3B) and functional richness (Fig. 3C) coincided in general terms with areas of high species richness, although with much variability. Southern Japan, Taiwan, the East and West coast of Australia, Southeast Africa, Southeast Brazil and the Southeast USA all showed high species richness, high endemism and functional richness. Some additional areas with high functional richness, like Southern Australia, Angola, North Chile and Western Continental Europe, only showed low to moderate species richness, however.

Using a hierarchical cluster analysis on all cells and species occurring in those cells, we identified 93 biogeographic units, which we defined as areas characterized by a unique community of shark species, ranging in size from 2 to 9645 one-degree cells (mean = 311 cells, see Methods for details). Most of these distinct shark communities were located on continental shelves and slopes (see Supporting Figure S1). Few biogeographic units covered large expanses in the open sea; those were mainly found in tropical areas.

Within each of these 93 biogeographic units, we then identified those cells with the highest (95^th^ percentile) species richness or endemicity in order to derive a structured framework for area prioritization. Based on these criteria we identified 4103 priority cells (14.2% of all cells) as possible areas for shark conservation (Fig. 3D), with the number of priority areas per biogeographic unit varying from 1 to 895 cells, or 5.1 to 67.7% (mean 15.3%) of each biogeographic unit's total number of cells. The biogeographic units with the highest number of priority areas were located in the open ocean, due to the large geographic extension of open-ocean biogeographic units (see Supporting Figure S1).

## Discussion

In this paper we have compiled and presented a global database of shark distribution for all species, and have derived global patterns of shark richness, endemicity and functional diversity. These patterns are based on a synthesis of all published information to date, and as such may help to inform marine conservation planning with respect to sharks. We have found that (1) shark species richness and endemicity are highest on continental shelves and at intermediate latitudes, (2) centers of shark species richness and endemicity tend to be geographically small, with only a few areas harbouring a disproportionately large species richness and endemicity, (3) total shark species richness, richness of finned species, endemicity and functional richness show some notable spatial differences, and (4) shark conservation may be difficult to pursue if based solely on a protected-area framework, due to the large spatial extent of conservation priority areas that represent all major biogeographic units; this is particularly true for the conservation of highly pelagic communities in the open ocean.

Based on our compilation of the available evidence, shark species richness was shown to peak at mid-latitudes, rather than at the tropics. A similar latitudinal pattern has been described for some highly oceanic groups, such as Foraminifera and tuna and billfishes, which show broad peaks of species richness between 20° and 30° in both hemispheres [24]–[27]. In tuna and billfishes, the causes of this pattern have been linked to optimal temperatures at those latitudes, in combination with the presence of highly productive frontal zones [25]. The pattern of species richness observed in sharks is different to that of Foraminifera and tunas and billfishes, however, in that it tends to peak at higher latitudes (30°–40°), and in coastal and shelf areas, rather than in the open oceans. Past research on marine fishes (mostly teleosts) has suggested the Philippines as a center of marine biodiversity [28]. In contrast, we found that shark species richness was lower in the Philippines compared to subtropical and temperate areas such as Taiwan, Southern Japan, and Eastern Australia. These differences could be due to different habitat requirements of sharks (as compared to teleost fishes) and the wider geographic scope of our study. Carpenter and Springer's [28] previous results agree with the present study in describing high diversity off Taiwan and Southeast Australia, which they attributed to the mixing of tropical and temperate faunas.

Individual hotspots of species richness and endemicity are concentrated in relatively small areas, yet basing a global strategy for shark conservation on these hotspots alone would leave many species without protection; this problem has already been noted in the terrestrial realm [4]. Including other aspects of biodiversity such as communities or ecosystems has been suggested as an alternative [4]. Our results indicate that a geographic approach to shark conservation that accounts for the biogeographic structure of unique shark communities would require much larger and more widespread areas than a classical hotspot approach that would only focus on the most species-rich cells.

Several caveats apply to our approach, which is necessarily based on existing, published knowledge. Most importantly, the existing knowledge is biased towards well-known species and regions. Our database reveals low data density and resulting increased uncertainty in the deep-sea and open ocean. Much more work is needed to resolve fine-scale distribution patterns in those habitats. We further note that our results should be applied cautiously with respect to reserve design. In addition to the abovementioned uncertainties we have not incorporated important ecological (e.g. dispersal and connectivity) or human-related variables (e.g. levels of exploitation) that may affect reserve design, and have not explicitly considered the costs and benefits of different conservation solutions. Nevertheless, our results do suggest that protected areas designed to conserve different shark communities would likely need to be very large, especially in oceanic waters. The inclusion of additional ecological criteria, such as connectivity among reserves, may lead to even larger priority areas, perhaps beyond the possibility of effective enforcement.

The evidence from our study suggests that conservation priority areas for species that are threatened by the burgeoning fin trade should be different than those for all shark species. Fin-trade species compared with all species tended to be concentrated more towards tropical regions, and towards open ocean waters. This finding is partly explained by a higher proportion of pelagic species in the fin trade, relative to all species, and supports the concept of using data from as many taxa and criteria as possible when designing conservation strategies [3]. From a conservation perspective it implies possible problems with enforcement of finning regulations in tropical countries with low management capacity, as well as on the high seas.

Furthermore we note that many areas with moderate species richness showed high functional richness, indicating that individual species may play unique ecological roles, and hence have low ecological redundancy. This means that different functions are fulfilled by few species, which may be important in maintaining the structure and function of some marine ecosystems [15].

In conclusion, we suggest that protecting the areas of high shark diversity found in our analysis could form part of a broader strategy to protect threatened marine species. The fact that these areas lie largely within coastal states' territorial waters, and that many of these states have good regulatory capacity (e.g. Australia, The United States, Japan) provides hope for more effective shark conservation, However, the large geographical extent of many priority areas that include both high richness and endemicity across representative biogeographic units casts doubt on the use of a global network of protected areas as a comprehensive strategy for shark conservation; the large extent of priority areas in international waters is especially problematic, since management and policing in those areas is more difficult to achieve compared with territorial waters. This problem extends particularly to fin-trade species which include a number of highly pelagic species. It is obvious that alternative approaches must form part of a comprehensive strategy to limit the fishing mortality of sensitive species, and halt the ongoing decline of shark populations [12], [ 19], [ 29]. We hope that the database that we have compiled can inform such a strategy, both at regional and global scales.

## Methods

### Data

The geographic distribution of each of 507 known shark species was extracted using available distributional data and expert knowledge from the scientific literature (from 1878 to present) and compiled in a Global Shark Distribution Database (Supporting Text S1, and Supporting Table S1 and Table S2). We included all species described and currently recognized by shark systematists, as well as species that are still unnamed but are already known to science. A detailed list of the species included and the supporting references for them can be found in the Supporting Information (Table S1 and Table S2). We used authoritative identification guides [e.g. 30]–[34] as a first approximation to the distribution of each species and obtained more precise limits with primary research papers, updated taxonomic reviews, reported range extensions, regional species lists and guides, and technical reports from fishery organizations (Table S2). If a species was regarded only as potentially present in a given area, or there were doubts about its real presence in a given area, we did not include that area in our maps. Thus, only cells with confirmed records of the species were included in the range maps. We also used point data from collection specimens included in the Global Biodiversity Information Facility (http://data.gbif.org), when these data were supported by a scientific publication. This information, however, is spatially non-homogenous and may vary in precision, as some areas (such as North America, Europe, or Australia) and species (such as commercial ones) are much better studied than others. Unfortunately, we could not standardize the database by research effort per unit area, due to the diverse nature of sources and sampling methods.

Published bathymetric limits were used to define the vertical distribution of each species; i.e. once the geographic limits of a species' distribution were identified, the vertical distribution limits were superimposed to identify the cells containing suitable habitat for the species to occur within the known range. This was done because depth is well known to strongly influence shark distribution and because bathymetric limits are known for a large majority of shark species, whereas the relative importance of other environmental variables (e.g. temperature, oxygen, bottom type) is much less resolved. For 59 widely distributed species that showed regional variation in their bathymetric limits (e.g. spiny dogfish *Squalus acanthias*, school shark *Galeorhinus galeus*), we partitioned the range map to accommodate the different depth limits. The distribution of species known only from a few individuals (less than 30 specimens) usually comprised actual records regardless of depth.

### Mapping

We used the R package PBSmapping [35] to build all maps. To map depth limits, bathymetric data were obtained from the well-known TOPEX topography database (http://topex.ucsd.edu) [36]. A grid of 1 degree latitude by 1 degree longitude was superimposed on each species' distribution map and one data point was recorded for each cell where the species was present. This particular scale was chosen because it approximates the spatial resolution of most distribution records that we reviewed, and because it is still fine enough to be useful in regional conservation planning exercises.

### Analysis

Species richness for each cell was obtained by summing the number of species occurring in each cell. We calculated species richness once for all species, and once for the 52 species that have been reported in the shark fin trade, as they may represent an urgent conservation priority due to high rates of exploitation [8], [9]. A species was included only if there was a scientific publication that documented its inclusion in the fin trade (Table S1). This list may not be complete, yet it represents our best knowledge of the fin trade based on published information. Endemism was estimated for each cell as the sum of the inverse of the range of the species present in that cell [21]. The range of a species was defined as the number of cells in which that species occurred.

Functional diversity was assessed by classifying the 507 shark species according to Compagno's [37] ecomorphotype description (Table S1). A particular ecomorphotype includes species, taxonomically related or not, that are similar in morphology, habitat and behaviour [37]. Consequently, areas with high richness of ecomorphotypes would have sharks performing more ecological functions than areas with low ecomorphotype diversity. We mapped the distribution of functional (ecomorphotype) richness as above.

In a further analysis, we identified shark biogeographic units in order to describe the distribution of unique shark communities and then obtained a grid of priority areas representative of all shark communities. Global shark biogeographic units were identified by using a hierarchical cluster analysis, a standard method used to find structure in a multivariate dataset. For this analysis, we used the Bray-Curtis dissimilarity index to group all 1°×1° cells according to their dissimilarity in the occurrence of shark species; clusters were identified at 70% dissimilarity [38]. The results were cells grouped together according to their similarity in shark species composition. In this way, we could identify 93 biogeographic units that corresponded to large-scale communities of similar species composition.

For each biogeographic unit, we identified the cells with the highest species richness or endemicity. These cells were defined as those having, at least, values of species richness or endemicity equal to the 95th percentile of the species richness or endemicity distribution of a given assemblage. In this way, we could identify the areas containing the highest number of species and the highest number of unique species for every shark biogeographic unit in the world in order to capture the largest number of species and unique species for every biogeographic unit.

## Supporting Information

Text S1 Introduction to the global shark database.
(PDF)

Table S1 Species list. It is indicated whether species are documented in the shark fin trade (FT), and which ecomorphotype (following Compagno 1990b) they represent.
(PDF)

Table S2 Data sources used in the mapping of shark species richness.
(XLS)

Figure S1 Global shark biogeographic units identified by a hierarchical cluster analysis on 507 shark species known to date. Different colours and patterns identify distinct biogeographic units. Biogeographic units are shown in different panels to improve visualization.
(TIF)
