# Supplementary material for: Global Diversity Hotspots and Conservation Priorities for Sharks
Source: PLoS One. 2011 May 5;6(5):e19356. doi: 10.1371/journal.pone.0019356 (PMC3088674; doi:10.1371/journal.pone.0019356)
Supplement: Text S1 — Introduction to the global shark database. (PDF) [file pone.0019356.s001.pdf]

Supplementary Information

Global Diversity Hotspots and Conservation Priorities for Sharks

Luis O. Lucifora[1,2,] Verónica B. García[2], Boris Worm[2]

[1] Consejo Nacional de Investigaciones Científicas y Técnicas (CONICET), Centro de Investigaciones Ecológicas Subtropicales (CIES), Centro de Investigaciones del Bosque Atlántico (CeIBA), Casilla de Correo 9, Puerto Iguazú, Misiones, N3370AVQ, Argentina

[2] Dalhousie University, Department of Biology, 1355 Oxford Street, Halifax, Nova Scotia, B3H 4J1, Canada

Contents:

Introduction: The Global Shark Distribution Database
Table S1: Species list
Table S2: Data sources used (as separate Excel File)
Figure S1: Shark biogeographic units

Introduction: The Global Shark Distribution Database

The Shark Distribution Database compiles the geographic distribution of all 507 shark species known to date. We exhaustively searched the scientific literature for the distribution ranges of the species, using authoritative identification guides, updated taxonomic reviews, range extensions, regional species lists and guides, and technical reports from fishery organizations (see data source file). Records published from 1878 to the present were incorporated in the database; consequently, this database represents the cumulative distribution of all known sharks.

To build this database, we mapped the geographic distribution of each species, taking into account latitudinal, longitudinal and depth limits, and superimposed a cell grid of 1 degree latitude by 1 degree longitude on the map. The values in the database represent the mean points of the cells in which the species is present, expressed in latitude and longitude.

We hope this database could be used by any research that is willing to expand the knowledge of ecology and conservation of chondrichthyan species. We strongly recommend contacting us if you are going to use the database; in this way, we will avoid overlap of projects (we have ongoing projects that require these data) and will stimulate collaborative projects.

Citation

Lucifora, L.O., García V.B., Worm, B. (2009) The Global Shark Distribution Database.

http://www.globalshark.ca/gs_distribution_db/data.php

Contact information:

Luis O. Lucifora, Consejo Nacional de Investigaciones Científicas y Técnicas, Centro de Investigaciones Ecológicas Subtropicales,
Centro de Investigaciones del Bosque Atlántico, Puerto Iguazú, Misiones, Argentina.
luis.lucifora@gmail.com

Verónica B. García, Department of Biology, Dalhousie University, Halifax, Nova Scotia, Canada.
vgarcia@mathstat.dal.ca

Acknowledgment

The Lenfest Ocean Program provided financial support to build this database.
