# Supplementary material for: Global Diversity Hotspots and Conservation Priorities for Sharks
Source: PLoS One. 2011 May 5;6(5):e19356. doi: 10.1371/journal.pone.0019356 (PMC3088674; doi:10.1371/journal.pone.0019356)
Supplement: Table S1 — Species list. It is indicated whether species are documented in the shark fin trade (FT), and which ecomorphotype (following Compagno 1990b) they represent. (PDF) [file pone.0019356.s002.pdf]

Table S1: Species list. It is indicated whether species are documented in the shark fin trade (FT), and which ecomorphotype (following Compagno 1990b) they represent.

Species name	FT	Ecomorphotype	Source for species included in the fin trade
Aculeola_nigra	no	bathic	
Alopias_pelagicus	yes	macroceanic	Clarke et al. 2006 (Ecol. Lett. 9:1115-1126)
Alopias_superciliosus	yes	macroceanic	Clarke et al. 2006 (Ecol. Lett. 9:1115-1126)
Alopias_vulpinus	yes	macroceanic	Clarke et al. 2006 (Ecol. Lett. 9:1115-1126)
Apristurus_albisoma	no	rhynchobathic	
Apristurus_ampliceps	no	rhynchobathic	
Apristurus_aphyodes	no	rhynchobathic	
Apristurus_australis	no	rhynchobathic	
Apristurus_brunneus	no	rhynchobathic	
Apristurus_bucephalus	no	rhynchobathic	
Apristurus_canutus	no	rhynchobathic	
Apristurus_exsanguis	no	rhynchobathic	
Apristurus_fedorovi	no	rhynchobathic	
Apristurus_gibbosus	no	rhynchobathic	
Apristurus_herklotsi	no	rhynchobathic	
Apristurus_indicus	no	rhynchobathic	
Apristurus_internatus	no	rhynchobathic	
Apristurus_investigatoris	no	rhynchobathic	
Apristurus_japonicus	no	rhynchobathic	
Apristurus_kampae	no	rhynchobathic	
Apristurus_laurussoni	no	rhynchobathic	
Apristurus_longicephalus	no	rhynchobathic	
Apristurus_macrorhynchus	no	rhynchobathic	
Apristurus_macrostomus	no	rhynchobathic	
Apristurus_manis	no	rhynchobathic	
Apristurus_melanoasper	no	rhynchobathic	
Apristurus_microps	no	rhynchobathic	
Apristurus_micropterygius	no	rhynchobathic	

Apristurus_nasutus	no	rhynchobathic
Apristurus_parvipinnis	no	rhynchobathic
Apristurus_pinguis	no	rhynchobathic
Apristurus_platyrhynchus	no	rhynchobathic
Apristurus_profundorum	no	rhynchobathic
Apristurus_riveri	no	rhynchobathic
Apristurus_saldanha	no	rhynchobathic
Apristurus_sibogae	no	rhynchobathic
Apristurus_sinensis	no	rhynchobathic
Apristurus_spongiceps	no	rhynchobathic
Apristurus_stenseni	no	rhynchobathic
Asymbolus_analis	no	leptobenthic
Asymbolus_funebris	no	leptobenthic
Asymbolus_galacticus	no	leptobenthic
Asymbolus_occiduus	no	leptobenthic
Asymbolus_pallidus	no	leptobenthic
Asymbolus_parvus	no	leptobenthic
Asymbolus_rubiginosus	no	leptobenthic
Asymbolus_submaculatus	no	leptobenthic
Asymbolus_vincenti	no	leptobenthic
Atelomycterus_baliensis	no	leptobenthic
Atelomycterus_fasciatus	no	leptobenthic
Atelomycterus_macleayi	no	leptobenthic
Atelomycterus_marmoratus	no	leptobenthic
Atelomycterus_marnkalha	no	leptobenthic
Aulohalaelurus_kanakorum	no	leptobenthic
Aulohalaelurus_labiosus	no	leptobenthic
Brachaelurus_colcloughi	no	probenthic
Brachaelurus_waddi	no	probenthic
Bythaelurus_alcocki	no	bathic
Bythaelurus_canescens	no	bathic
Bythaelurus_clevai	no	bathic

Bythaelurus_dawsoni	no	bathic	
Bythaelurus_hispidus	no	bathic	
Bythaelurus_immaculatus	no	bathic	
Bythaelurus_incanus	no	bathic	
Bythaelurus_lutarius	no	bathic	
Bythaelurus_spB	no	bathic	
Carcharhinus_acronotus	no	littoral	
Carcharhinus_albimarginatus	yes	eurytrophic	Compagno et al. 2005 (Sharks of the World, Collins, London), Anderson & Ahmed 1993 (The Shark Fisheries in the Maldives, Ministry of Fisheries and Agriculture of the Maldives and FAO, Male)
Carcharhinus_altimus	yes	eurytrophic	Anderson & Ahmed 1993 (The Shark Fisheries in the Maldives, Ministry of Fisheries and Agriculture of the Maldives and FAO, Male)
Carcharhinus_amblyrhynchoides	yes	littoral	Last & Stevens 2009 (Sharks and Rays of Australia, 2nd edition, Harvard University Press, Cambridge)
Carcharhinus_amblyrhynchos	yes	littoral	Last & Stevens 2009 (Sharks and Rays of Australia, 2nd edition, Harvard University Press, Cambridge)
Carcharhinus_amboinensis	yes	eurytrophic	Last & Stevens 2009 (Sharks and Rays of Australia, 2nd edition, Harvard University Press, Cambridge)
Carcharhinus_borneensis	no	littoral	
Carcharhinus_brachyurus	yes	eurytrophic	L.O. Lucifora (pers. Obs.)
Carcharhinus_brevipinna	yes	eurytrophic	Last & Stevens 2009 (Sharks and Rays of Australia, 2nd edition, Harvard University Press, Cambridge)
Carcharhinus_cautus	no	littoral	
Carcharhinus_dussumieri	yes	littoral	Last & Stevens 2009 (Sharks and Rays of Australia, 2nd edition, Harvard University Press, Cambridge)
Carcharhinus_falciformis	yes	macroceanic	Clarke et al. 2006 (Ecol. Lett. 9:1115-1126)
Carcharhinus_fitzroyensis	no	littoral	
Carcharhinus_galapagensis	yes	eurytrophic	Dalzell et al. 2008 (in: Sharks of the Open Ocean, Camhi et al. Eds, pp. 268-274, Blackwell, Oxford)
Carcharhinus_hemiodon	no	littoral	
Carcharhinus_isodon	no	littoral	
Carcharhinus_leiodon	no	littoral	
Carcharhinus_leucas	yes	eurytrophic	Clarke et al. 2006 (Ecol. Lett. 9:1115-1126)
Carcharhinus_limbatus	yes	eurytrophic	Compagno et al. 2005 (Sharks of the World, Collins, London), Anderson & Ahmed 1993 (The Shark Fisheries in the Maldives, Ministry of Fisheries and Agriculture of the Maldives and FAO, Male), White et al. 2006 (Economically Important Sharks and Rays of Indonesia, Australian Centre for International Agricultural Research, Canberra)
Carcharhinus_longimanus	yes	macroceanic	Clarke et al. 2006 (Ecol. Lett. 9:1115-1126)
Carcharhinus_macloti	yes	littoral	White et al. 2006 (Economically Important Sharks and Rays of Indonesia, Australian Centre for International Agricultural Research, Canberra)
Carcharhinus_melanopterus	yes	littoral	White et al. 2006 (Economically Important Sharks and Rays of Indonesia, Australian Centre for International Agricultural Research, Canberra), Anderson & Ahmed 1993 (The Shark Fisheries in the Maldives, Ministry of Fisheries and Agriculture of the Maldives and FAO, Male)
Carcharhinus_obscurus	yes	eurytrophic	Clarke et al. 2006 (Ecol. Lett. 9:1115-1126)

Carcharhinus_perezi	yes	eurytrophic	Compagno et al. 2005 (Sharks of the World, Collins, London)
Carcharhinus_plumbeus	yes	eurytrophic	Clarke et al. 2006 (Ecol. Lett. 9:1115-1126)
Carcharhinus_porosus	no	littoral	
Carcharhinus_sealei	yes	littoral	White et al. 2006 (Economically Important Sharks and Rays of Indonesia, Australian Centre for International Agricultural Research, Canberra)
Carcharhinus_signatus	yes	eurytrophic	Santana et al. 2009 (In: The Conservation Status of Pelagic Sharks and Rays, Camhi et al. Eds., IUCN, Oxford)
Carcharhinus_sorrah	yes	littoral	Last & Stevens 2009 (Sharks and Rays of Australia, 2nd edition, Harvard University Press, Cambridge)
Carcharhinus_spA	no	littoral	
Carcharhinus_tilstoni	no	littoral	
Carcharias_taurus	yes	eurytrophic	L.O. Lucifora (pers. Obs.)
Carcharodon_carcharias	yes	archipelagic	Clarke 2004 (Shark Products Trade in Hong Kong and Mainland China and Implementation of the CITES Shark. Listings. TRAFFIC East Asia, Hong Kong.)
Centrophorus_acus	no	bathic	
Centrophorus_atromarginatus	no	bathic	
Centrophorus_granulosus	no	bathic	
Centrophorus_harrissoni	no	bathic	
Centrophorus_isodon	no	bathic	
Centrophorus_lusitanicus	no	bathic	
Centrophorus_moluccensis	no	bathic	
Centrophorus_niaukang	no	bathic	
Centrophorus_seychellorum	no	bathic	
Centrophorus_spA	no	bathic	
Centrophorus_squamosus	no	bathic	
Centrophorus_tesselatus	no	bathic	
Centrophorus_westraliensis	no	bathic	
Centrophorus_zeehaani	no	bathic	
Centroscyllium_excelsum	no	bathic	
Centroscyllium_fabricii	no	bathic	
Centroscyllium_granulatum	no	bathic	
Centroscyllium_kamoharai	no	bathic	
Centroscyllium_nigrum	no	bathic	
Centroscyllium_ornatum	no	bathic	
Centroscyllium_ritteri	no	bathic	
Centroscymnus_coelolepis	no	bathic	

Centroscymnus_owstoni	no	bathic	
Centroselachus_crepidater	no	bathic	
Cephaloscyllium_albipinnum	no	probenthic	
Cephaloscyllium_circulopullum	no	probenthic	
Cephaloscyllium_cooki	no	probenthic	
Cephaloscyllium_fasciatum	no	probenthic	
Cephaloscyllium_hiscosellum	no	probenthic	
Cephaloscyllium_isabellum	no	probenthic	
Cephaloscyllium_laticeps	no	probenthic	
Cephaloscyllium_maculatum	no	probenthic	
Cephaloscyllium_pardelotum	no	probenthic	
Cephaloscyllium_pictum	no	probenthic	
Cephaloscyllium_sarawakensis	no	probenthic	
Cephaloscyllium_signourum	no	probenthic	
Cephaloscyllium_silasi	no	probenthic	
Cephaloscyllium_sp_Phillipines	no	probenthic	
Cephaloscyllium_sp_Sokotra	no	probenthic	
Cephaloscyllium_speccum	no	probenthic	
Cephaloscyllium_sufflans	no	probenthic	
Cephaloscyllium_umbratile	no	probenthic	
Cephaloscyllium_variegatum	no	probenthic	
Cephaloscyllium_ventriosum	no	probenthic	
Cephaloscyllium_zebrum	no	probenthic	
Cephalurus_cephalus	no	anoxybathic	
Cephalurus_spA		anoxybathic	
Cetorhinus_maximus	yes	tachypelagic_filter	Compagno et al. 2005 (Sharks of the World, Collins, London)
Chaenogaleus_macrostoma	no	littoral	
Chiloscyllium_arabicum	no	leptobenthic	
Chiloscyllium_burmensis	no	leptobenthic	
no			
Chiloscyllium_griseum	no	leptobenthic	
Chiloscyllium_hasselti	no	leptobenthic	
Chiloscyllium_indicum	no	leptobenthic	

Chiloscyllium_plagiosum	no	leptobenthic
Chiloscyllium_punctatum	no	leptobenthic
Chlamydoselachus_anguineus	no	anguilloid
Chlamydoselachus_spA	no	anguilloid
Cirrhigaleus_asper	no	littoral
Cirrhigaleus_australis	no	littoral
Cirrhigaleus_barbifer	no	littoral
Cirrhoscyllium_expolitum	no	leptobenthic
Cirrhoscyllium_formosanum	no	leptobenthic
Cirrhoscyllium_japonicum	no	leptobenthic
Ctenacis_fehlmanni	no	littoral
Dalatias_licha	no	bathic
Deania_calcea	no	rhynchobathic
Deania_hystricosa	no	rhynchobathic
Deania_profundorum	no	rhynchobathic
Deania_quadrispinosum	no	rhynchobathic
Echinorhinus_brucus	no	bathic
Echinorhinus_cookei	no	bathic
Eridacnis_barbouri	no	leptobenthic
Eridacnis_radcliffei	no	leptobenthic
Eridacnis_sinuans	no	leptobenthic
Etmopterus_baxteri	no	bathic
Etmopterus_bigelowi	no	bathic
Etmopterus_brachyurus	no	bathic
Etmopterus_bullisi	no	bathic
Etmopterus_burgessi	no	bathic
Etmopterus_carteri	no	bathic
Etmopterus_caudistigmus	no	bathic
Etmopterus_decacuspidatus	no	bathic
Etmopterus_dianthus	no	bathic
Etmopterus_dislineatus	no	bathic
Etmopterus_evansi	no	bathic

Etmopterus_fusus	no	bathic	
Etmopterus_gracilispinis	no	bathic	
Etmopterus_granulosus	no	bathic	
Etmopterus_hillianus	no	bathic	
Etmopterus_litvinovi	no	bathic	
Etmopterus_lucifer	no	bathic	
Etmopterus_molleri	no	bathic	
Etmopterus_perryi	no	bathic	
Etmopterus_polli	no	bathic	
Etmopterus_princeps	no	bathic	
Etmopterus_pseudosqualiolus	no	bathic	
Etmopterus_pusillus	no	bathic	
Etmopterus_pycnolepis	no	bathic	
Etmopterus_robinsi	no	bathic	
Etmopterus_schultzi	no	bathic	
Etmopterus_sentosus	no	bathic	
Etmopterus_spA	no	bathic	
Etmopterus_spinax	no	bathic	
Etmopterus_splendidus	no	bathic	
Etmopterus_unicolor	no	bathic	
Etmopterus_villosus	no	bathic	
Etmopterus_virens	no	bathic	
Eucrossorhinus_dasypogon	no	squatinobenthic	
Euprotomicroides_zantedeschia	no	microceanic	
Euprotomicrus_bispinatus	no	microceanic	
Eusphyra_blochii	no	sphyrnid	
Figaro_boardmani	no	probenthic	
Figaro_striatus	no	probenthic	
Furgaleus_macki	no	teuthitrophic	
Galeocerdo_cuvier	yes	eurytrophic	Clarke et al. 2006 (Ecol. Lett. 9:1115-1126)
Galeorhinus_galeus	yes	littoral	Compagno et al. 2005 (Sharks of the World, Collins, London)
Galeus_antillensis		probenthic	

no

Galeus_arae	no	probenthic
Galeus_atlanticus	no	probenthic
Galeus_cadenati	no	probenthic
Galeus_eastmani	no	probenthic
Galeus_gracilis	no	probenthic
Galeus_longirostris	no	probenthic
Galeus_melastomus	no	probenthic
Galeus_mincaronei	no	probenthic
Galeus_murinus	no	probenthic
Galeus_nipponensis	no	probenthic
Galeus_piperatus	no	probenthic
Galeus_polli	no	probenthic
Galeus_priapus	no	probenthic
Galeus_sauteri	no	probenthic
Galeus_schultzi	no	probenthic
Galeus_sp1	no	probenthic
Galeus_springeri	no	probenthic
GenusA_spA	no	bathic
Ginglymostoma_cirratum	no	littoral
Glyphis_gangeticus	no	eurytrophic
Glyphis_garricki	no	eurytrophic
Glyphis_glyphis	no	eurytrophic
Glyphis_siamensis	no	eurytrophic
Glyphis_spB	no	eurytrophic
Gogolia_filewoodi	no	probenthic
Gollum_attenuatus	no	bathic
Gollum_spA	no	bathic
Gollum_spB	no	bathic
Halaelurus_boesemani	no	leptobenthic
Halaelurus_buergeri	no	leptobenthic
Halaelurus_lineatus	no	leptobenthic
Halaelurus_maculosus	no	leptobenthic

Halaelurus_natalensis	no	leptobenthic	
Halaelurus_quagga	no	leptobenthic	
Halaelurus_sellus	no	leptobenthic	
Haploblepharus_edwardsi	no	probenthic	
Haploblepharus_fuscus	no	probenthic	
Haploblepharus_kistnasamyi	no	probenthic	
Haploblepharus_pictus	no	probenthic	
Hemicyllium_galei	no	leptobenthic	
Hemicyllium_henryi	no	leptobenthic	
Hemigaleus_australiensis	no	teuthitrophic	
Hemigaleus_microstoma	no	teuthitrophic	
Hemipristis_elongatus	yes	littoral	Last & Stevens 2009 (Sharks and Rays of Australia, 2nd edition, Harvard University Press, Cambridge)
Hemiscyllium_freycineti	no	leptobenthic	
Hemiscyllium_hallstromi	no	leptobenthic	
Hemiscyllium_ocellatum	no	leptobenthic	
Hemiscyllium_strahani	no	leptobenthic	
Hemiscyllium_trispeculare	no	leptobenthic	
Hemitriakis_abdita	no	littoral	
Hemitriakis_complicofasciata	no	littoral	
Hemitriakis_falcata	no	littoral	
Hemitriakis_indroyonoi	yes	littoral	White et al. 2006 (Economically Important Sharks and Rays of Indonesia, Australian Centre for International Agricultural Research, Canberra)
Hemitriakis_japanica	no	littoral	
Hemitriakis_leucoperiptera	no	littoral	
Hemitriakis_spA	no	littoral	
Heptranchias_perlo	no	bathic	
Heterodontus_francisci	no	probenthic	
Heterodontus_galeatus	no	probenthic	
Heterodontus_japonicus	no	probenthic	
Heterodontus_mexicanus	no	probenthic	
Heterodontus_omanensis	no	probenthic	
Heterodontus_portusjacksoni	no	probenthic	
Heterodontus_quoyi	no	probenthic	

Heterodontus_ramalheira	no	probenthic	
Heterodontus_zebra	no	probenthic	
Heteroscymnoides_marleyi	no	microceanic	
Hexanchus_griseus	no	eurytrophic	
Hexanchus_nakamurai	no	bathic	
Holohalaelurus_favus	no	leptobenthic	
Holohalaelurus_grennian	no	leptobenthic	
Holohalaelurus_melanostigma	no	leptobenthic	
Holohalaelurus_punctatus	no	leptobenthic	
Holohalaelurus_regani	no	leptobenthic	
Hypogaleus_hyugaensis	no	littoral	
Iago_garricki	no	littoral	
Iago_omanensis	no	anoxybathic	
Iago_spA	no	anoxybathic	
Isistius_brasiliensis	no	microceanic	
Isistius_labialis	no	microceanic	
Isistius_plutodus	no	microceanic	
Isogomphodon_oxyrhynchus	no	rhynchobathic	
Isurus_oxyrinchus	yes	tachypelagic	Clarke et al. 2006 (Ecol. Lett. 9:1115-1126)
Isurus_paucus	yes	tachypelagic	Last & Stevens 2009 (Sharks and Rays of Australia, 2nd edition, Harvard University Press, Cambridge)
Lamiopsis_temmincki	no	littoral	
Lamna_ditropis	yes	tachypelagic	Camhi et al. 2008 (In: Sharks of the Open Ocean, pp. 166-192, Blackwell, London)
Lamna_nasus	yes	tachypelagic	Last & Stevens 2009 (Sharks and Rays of Australia, 2nd edition, Harvard University Press, Cambridge)
Leptocharias_smithii	no	littoral	
Loxodon_macrorhinus	yes	littoral	White et al. 2006 (Economically Important Sharks and Rays of Indonesia, Australian Centre for International Agricultural Research, Canberra)
Megachasma_pelagios	no	macroceanic_filter	
Miroscyllium_sheikoi	no	bathic	
Mitsukurina_owstoni	no	rhynchobathic	
Mollisquama_parini	no	bathic	
Mustelus_albipinnis	no	cancritrophic	
Mustelus_antarcticus	no	cancritrophic	
Mustelus_asterias	no	cancritrophic	

Mustelus_californicus	no	cancritrophic	
Mustelus_canis	no	cancritrophic	
Mustelus_dorsalis	no	cancritrophic	
Mustelus_fasciatus	no	cancritrophic	
Mustelus_griseus	no	cancritrophic	
Mustelus_henlei	no	cancritrophic	
Mustelus_higmani	no	cancritrophic	
Mustelus_lenticulatus	no	cancritrophic	
Mustelus_lunulatus	no	cancritrophic	
Mustelus_manazo	no	cancritrophic	
Mustelus_mento	no	cancritrophic	
Mustelus_minicanis	no	cancritrophic	
Mustelus_mosis	no	cancritrophic	
Mustelus_mustelus	no	cancritrophic	
Mustelus_norrisi	no	cancritrophic	
Mustelus_palumbes	no	cancritrophic	
Mustelus_punctulatus	no	cancritrophic	
Mustelus_ravidus	no	cancritrophic	
Mustelus_schmitti	no	cancritrophic	
Mustelus_sinusmexicanus	no	cancritrophic	
Mustelus_sp1	no	cancritrophic	
Mustelus_sp2	no	cancritrophic	
Mustelus_sp3	no	cancritrophic	
Mustelus_stevensi	no	cancritrophic	
Mustelus_walkeri	no	cancritrophic	
Mustelus_whitneyi	no	cancritrophic	
Mustelus_widodoi	no	cancritrophic	
Nasolamia_velox	yes	littoral	Whoriskey (Bsc Thesis, Dalhousie University, Halifax)
Nebrius_ferrugineus	no	littoral	
Negaprion_acutidens	yes	eurytrophic	Last & Stevens 2009 (Sharks and Rays of Australia, 2nd edition, Harvard University Press, Cambridge)
Negaprion_brevirostris	yes	eurytrophic	Compagno 2002 (In: The Living Marine Resources of the Western Central Atlantic, Vol. 1, FAO, Rome)
Notorynchus_cepedianus	no	eurytrophic	

Odontaspis_ferox	yes	bathic	Anderson & Ahmed 1993 (The Shark Fisheries in the Maldives, Ministry of Fisheries and Agriculture of the Maldives and FAO, Male), Last & Stevens 2009 (Sharks and Rays of Australia, 2nd edition, Harvard University Press, Cambridge)
Odontaspis_noronhai	yes	bathic	Amorim et al. 1998 (Mar. Freshw. Res. 49:621-632)
Orectolobus_floridus	no	squatinobenthic	
Orectolobus_halei	no	squatinobenthic	
Orectolobus_hutchinsi	no	squatinobenthic	
Orectolobus_japonicus	no	squatinobenthic	
Orectolobus_maculatus	no	squatinobenthic	
Orectolobus_ornatus	no	squatinobenthic	
Orectolobus_parvimaculatus	no	squatinobenthic	
Orectolobus_reticulatus	no	squatinobenthic	
Orectolobus_wardi	no	squatinobenthic	
Oxynotus_bruniensis	no	bathic	
Oxynotus_caribbaeus	no	bathic	
Oxynotus_centrina	no	bathic	
Oxynotus_japonicus	no	bathic	
Oxynotus_paradoxus	no	bathic	
Paragaleus_leucolomatus	no	littoral	
Paragaleus_pectoralis	no	teuthitrophic	
Paragaleus_randalli	no	littoral	
Paragaleus_tengi	no	littoral	
Parascyllium_collare	no	leptobenthic	
Parascyllium_elongatum	no	leptobenthic	
Parascyllium_ferrugineum	no	leptobenthic	
Parascyllium_sparsimaculatum	no	leptobenthic	
Parascyllium_variolatum	no	leptobenthic	
Parmaturus_albimarginatus	no	bathic	
Parmaturus_albipenis	no	bathic	
Parmaturus_bigus	no	bathic	
Parmaturus_campechiensis	no	bathic	
Parmaturus_lanatus	no	bathic	
Parmaturus_macmillani	no	bathic	
Parmaturus_melanobranchius	no	bathic	

Parmaturus_pilosus	no	bathic	
Parmaturus_xaniurus	no	anoxybathic	
Pentanchus_profundicolus	no	rhynchobathic	
Pliotrema_warreni	no	pristobenthic	
Poroderma_africanum	no	probenthic	
Poroderma_pantherinum	no	probenthic	
Prionace_glauca	yes	macroceanic	Clarke et al. 2006 (Ecol. Lett. 9:1115-1126)
Pristiophorus_cirratus	no	pristobenthic	
Pristiophorus_delicatus	no	pristobenthic	
Pristiophorus_japonicus	no	pristobenthic	
Pristiophorus_nudipinnis	no	pristobenthic	
Pristiophorus_schroederi	no	pristobenthic	
Pristiophorus_spC	no	pristobenthic	
Pristiophorus_spD	no	pristobenthic	
Proscyllium_habereri	no	leptobenthic	
Proscyllium_magnificum	no	leptobenthic	
Proscymnodon_macracanthus	no	bathic	
Proscymnodon_plunketi	no	bathic	
Pseudocarcharias_kamoharai	no	microceanic	
Pseudoginglymostoma_brevicaudatum	yes	littoral	Nel et al. 2004 (IUCN Red List, IUCN)
Pseudotriakis_microdon	no	bathic	
Rhincodon_typus	yes	macroceanic_filter	Clarke 2004 (Shark Products Trade in Hong Kong and Mainland China and Implementation of the CITES Shark. Listings. TRAFFIC East Asia, Hong Kong)
Rhizoprionodon_acutus	yes	littoral	White et al. 2006 (Economically Important Sharks and Rays of Indonesia, Australian Centre for International Agricultural Research, Canberra)
Rhizoprionodon_lalandii	no	littoral	
Rhizoprionodon_longurio	no	littoral	
Rhizoprionodon_oligolinx	yes	littoral	White et al. 2006 (Economically Important Sharks and Rays of Indonesia, Australian Centre for International Agricultural Research, Canberra)
Rhizoprionodon_porosus	no	littoral	
Rhizoprionodon_taylori	yes	littoral	White et al. 2006 (Economically Important Sharks and Rays of Indonesia, Australian Centre for International Agricultural Research, Canberra)
Rhizoprionodon_terraenovae	no	littoral	
Schroederichthys_bivius	no	leptobenthic	
Schroederichthys_chilensis	no	leptobenthic	

Schroederichthys_maculatus	no	leptobenthic
Schroederichthys_saurisqualus	no	leptobenthic
Schroederichthys_tenuis	no	leptobenthic
Scoliodon_laticaudus	no	littoral
Scyliorhinus_boa	no	probenthic
Scyliorhinus_canicula	no	probenthic
Scyliorhinus_capensis	no	probenthic
Scyliorhinus_cervigoni	no	probenthic
Scyliorhinus_comoroensis	no	probenthic
Scyliorhinus_garmani	no	probenthic
Scyliorhinus_haeckelii	no	probenthic
Scyliorhinus_hesperius	no	probenthic
Scyliorhinus_meadi	no	probenthic
Scyliorhinus_retifer	no	probenthic
Scyliorhinus_spA	no	probenthic
Scyliorhinus_spB	no	probenthic
Scyliorhinus_stellaris	no	probenthic
Scyliorhinus_tokubee	no	probenthic
Scyliorhinus_torazame	no	probenthic
Scyliorhinus_torrei	no	probenthic
Scylliogaleus_quecketti	no	cancritrophic
Scymnodalatias_albicauda	no	bathic
Scymnodalatias_garricki	no	bathic
Scymnodalatias_oligodon	no	bathic
Scymnodalatias_sherwoodi	no	bathic
Scymnodon_ringens	no	bathic
Somniosus_antarcticus	no	eurytrophic
Somniosus_longus	no	bathic
Somniosus_microcephalus	no	eurytrophic
Somniosus_pacificus	no	eurytrophic
Somniosus_rostratus	no	bathic
Somniousus_spA	no	bathic

Sphyrna_corona			sphyrnid	
Sphyrna_lewini		yes	sphyrnid	Clarke et al. 2006 (Ecol. Lett. 9:1115-1126)
Sphyrna_media		no	sphyrnid	
Sphyrna_mokarran		yes	sphyrnid	Clarke et al. 2006 (Ecol. Lett. 9:1115-1126)
Sphyrna_spA	no	yes	sphyrnid	Quattro et al. 2006 (Mar. Biol. 148: 1143-1155)
Sphyrna_tiburo		no	sphyrnid_cancritrophic	
Sphyrna_tudes		no	sphyrnid	
Sphyrna_zygaena		yes	sphyrnid	Clarke et al. 2006 (Ecol. Lett. 9:1115-1126)
Squaliolus_aliae			microceanic	
Squaliolus_laticaudus		no	microceanic	
Squalus_acanthias		no	littoral	
Squalus_albifrons		no	littoral	
Squalus_altipinnis		no	littoral	
Squalus_blainvillei	no	no	littoral	
Squalus_bucephalus		no	littoral	
Squalus_chloroculus		no	littoral	
Squalus_crassispinus		no	littoral	
Squalus_cubensis		no	littoral	
Squalus_edmundsi		no	littoral	
Squalus_grahami		no	littoral	
Squalus_griffini		no	littoral	
Squalus_hemipinnis		no	littoral	
Squalus_japonicus		no	littoral	
Squalus_lalannei		no	littoral	
Squalus_megalops		no	littoral	
Squalus_melanurus		no	littoral	
Squalus_mitsukurii		no	littoral	
Squalus_montalbani		no	littoral	
Squalus_nasutus		no	littoral	
Squalus_notocaudatus		no	littoral	
Squalus_rancureli		no	littoral	
Squalus_raulensis		no	littoral	

Squalus_sp1	no	littoral	
Squalus_spA	no	littoral	
Squalus_spB	no	littoral	
Squatina_aculeata	no	squatinobenthic	
Squatina_africana	no	squatinobenthic	
Squatina_albipunctata	no	squatinobenthic	
Squatina_argentina	no	squatinobenthic	
Squatina_armata	no	squatinobenthic	
Squatina_australis	no	squatinobenthic	
Squatina_californica	no	squatinobenthic	
Squatina_dumeril	no	squatinobenthic	
Squatina_formosa	no	squatinobenthic	
Squatina_guggenheim	no	squatinobenthic	
Squatina_heteroptera	no	squatinobenthic	
Squatina_japonica	no	squatinobenthic	
Squatina_legnota	no	squatinobenthic	
Squatina_mexicana	no	squatinobenthic	
Squatina_nebulosa	no	squatinobenthic	
Squatina_occulta	no	squatinobenthic	
Squatina_oculata	no	squatinobenthic	
Squatina_pseudocellata	no	squatinobenthic	
Squatina_squatina	no	squatinobenthic	
Squatina_tergocellata	no	squatinobenthic	
Squatina_tergocellatoides	no	squatinobenthic	
Stegostoma_fasciatum	no	littoral	
Sutorectus_tentaculatus	no	squatinobenthic	
Triaenodon_obesus	yes	littoral	Last & Stevens 2009 (Sharks and Rays of Australia, 2nd edition, Harvard University Press, Cambridge)
Triakis_acutipinna	no	littoral	
Triakis_maculata	no	littoral	
Triakis_megalopterus	no	littoral	
Triakis_scyllium	no	littoral	
Triakis_semifasciata	no	littoral	

Trigonognathus_kabeyai	no	bathic
Zameus_ichiharai	no	bathic
Zameus_squamulosus	no	bathic
Zameus_squamulosus	no	bathic
