# Supplementary figures and images for: Global Diversity Hotspots and Conservation Priorities for Sharks
Source: PLoS One. 2011 May 5;6(5):e19356. doi: 10.1371/journal.pone.0019356 (PMC3088674; doi:10.1371/journal.pone.0019356)

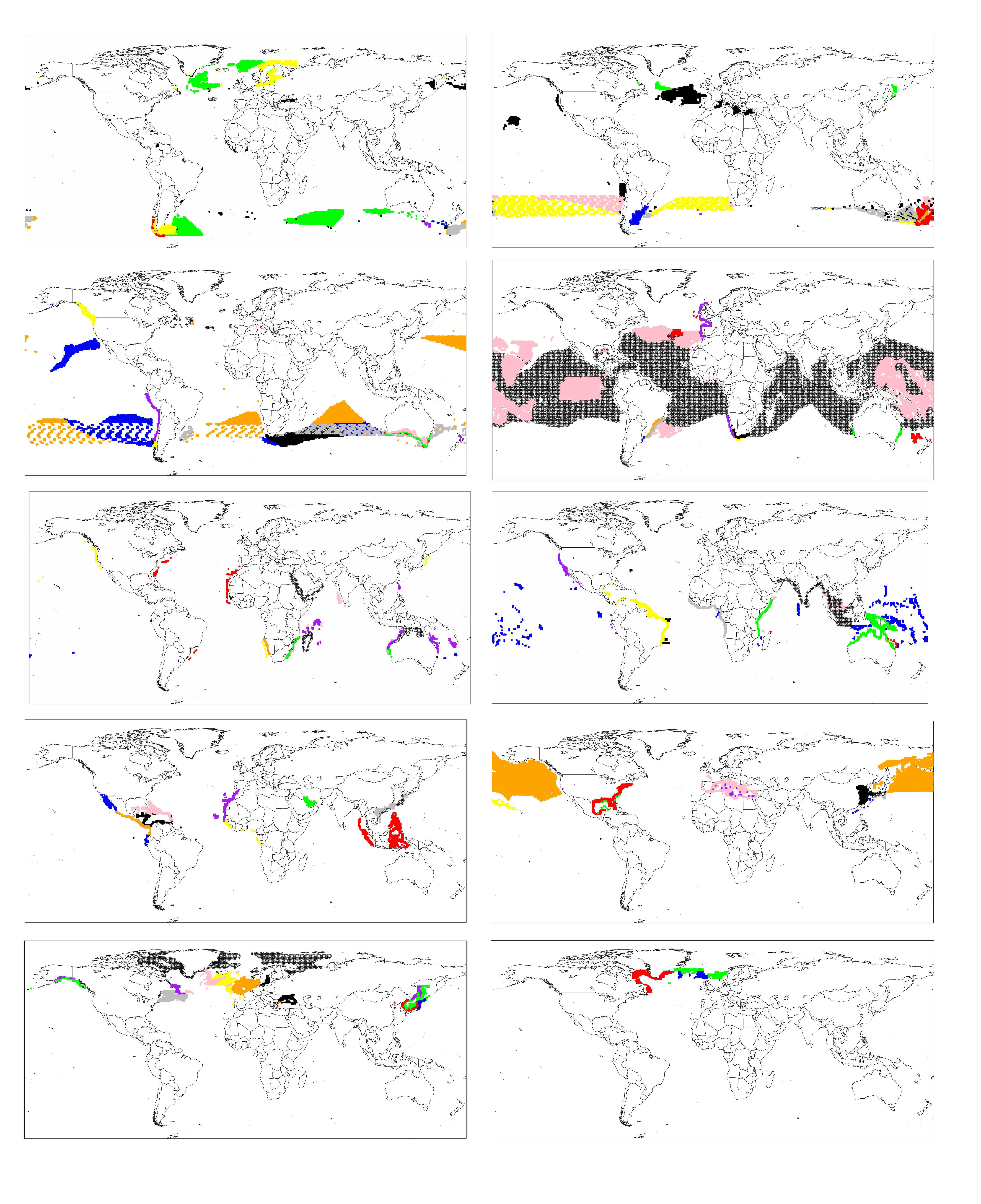

Supplement: Figure S1 — Global shark biogeographic units identified by a hierarchical cluster analysis on 507 shark species known to date. Different colours and patterns identify distinct biogeographic units. Biogeographic units are shown in different panels to improve visualization. (TIF) [file pone.0019356.s004.tif]
